# Female reproductive potential after oncological treatment: a rare case report of acute myeloid leukemia in monozygotic twin sisters with literature review

**DOI:** 10.1186/s13048-019-0607-0

**Published:** 2020-01-03

**Authors:** Tanja Burnik Papler, Eda Vrtacnik Bokal, Nina Jančar

**Affiliations:** Department of Human Reproduction, Division of Obstetrics and Gynecology, University Medical Center Ljubljana, Ljubljana, Slovenia

**Keywords:** Cancer treatment, Premature ovarian failure, Fertility preservation, Twins, Acute myeloid leukemia

## Abstract

**Background:**

Acute myeloid leukemia (AML) in monozygotic twins is a rare event and, until now, only a few cases have been reported. Due to improved oncological treatment and cancer survival rates, quality of life considerations post-treatment have become an important aspect in the treatment regime. The ability to have their own biological children is considered one of the most important indicators of quality of life by cancer survivors. As fertility following oncological treatment is often impaired, fertility preservation methods should be offered to these patients prior to the treatment. Here, we present a very rare case in which we can in vivo observe the impact of oncological treatment on female fertility when applied before and after puberty.

**Case presentation:**

This is a very rare case of concordant AML in monozygotic twin sisters. Twin A became sick at the age of 21 months. She was treated with cytostatic medications and then underwent bone marrow transplantation (BMT), the donor being her twin sister B. After 27 years, she is disease free and has regular periods. After trying to conceive for 4 years, she was seen by an infertility specialist. She underwent hysteroscopic uterine septum removal and laparoscopic enucleation of bilateral paraovarian cysts. Following those procedures, she immediately conceived naturally. Twin B became sick at 15 years of age. She was treated with chemotherapy and cranial radiation and relapsed after 10 years. She then received chemotherapy and had a BMT. Until relapse, she had normal menstrual cycles. After the second treatment she became amenorrhoeic and is now part of an oocyte donation program.

**Conclusions:**

This is a case of AML in monozygotic twins who, after treatment, have completely different reproductive potential. They both received oncological treatment, and one of them conceived conceived naturally while the other suffered premature ovarian failure and is not able to have a biological child. Based on the outcome of this case, it appears that the pre-pubertal state truly serves as protection against ovarian failure.

## Background

Concordant leukemia in monozygotic (MZ) twins was first described in 1882 [1], and since then, few cases have been reported [2]. The interval between disease onset in each twin varies from a few months to a few years [2]. Also, the genetics of childhood acute leukemia is highly variable [3] and germline mutations in the *C/EBPA* gene have already been described [4].

Due to improvements in treatment, life expectancy of cancer patients has become longer in the past decades, and for this reason, their quality of life once the treatment is complete is becoming increasingly important [5]. A chance to have one’s own biological children after treatment is an important aspect of quality of life. It is well known that cancer treatment can cause subfertility or infertility; therefore, methods for fertility preservation prior to oncological treatment are crucial to keep reproductive potential after concluding the treatment.

We present a rare case of MZ twin sisters who both developed AML and share the same germline and somatic *C/EBPA* gene mutation but have different reproductive potential after oncological treatment.

## Case presentation

This is a case of monozygotic twins. Twin sister A was diagnosed with AML at the age of 21 months. GTG banding revealed a normal karyotype. She was medicated with cytostatic medications according to the AML-BFM 83 protocol [6]. As there were no malignant cells found in the cerebrospinal fluid, she underwent no radiotherapy. She did receive a bone marrow transplantation (BMT), with the donor tissue coming from her twin sister. Following the BMT, she was disease-free. Her menarche was at 11 years, but her menstruation became irregular, prolonged and heavier around the age of 15. She received progestogens and, with time, her menstrual cycle normalized. At 26 years she was seen at the reproductive medicine unit for primary infertility. Until that time, she had been trying to conceive for 4 years. Her gynecological examination was normal. However, transvaginal ultrasound revealed a low antral follicle count with only 2 and 3 antral follicles seen on the right and the left ovary, respectively. Uterine septum and bilateral para-ovarian cysts were also seen via ultrasound. Her blood hormone results were as follows: FSH 5.6 IU/L, LH 1.3 IU/L. Hysteroscopic uterine septum resection and laparoscopic removal of bilateral paraovarian cysts was performed. After the surgery, she conceived naturally. Gestational diabetes, which was discovered with the oral glucose tolerance test performed in the 27th week of pregnancy, was treated with insulin. At 40 weeks she gave birth vaginally to a healthy baby girl. The child’s birth weight was 2530 g and the Apgar score was 9/9. She tested negative for the *C/EBPA* mutation. According to the patient: A double miracle had happened – she naturally conceived and delivered a child without the *C/EBPA* mutation.

Twin sister B was diagnosed with AML at the age of 15 and was treated with cytostatic drugs according to the AML-BFM 98 protocol [7]. She then received a total dose of 12 Gy by cranial radiation. She also had a normal karyotype. However, at the time of the diagnosis in twin B, advances in molecular genetics resulted in the discovery of the germline N-terminal frameshift mutation in the *C/EBPA* gene in both sisters [8]. They both had an identical somatic C-terminal mutation, and twin A had an additional somatic C-terminal mutation *C/EBPA* mutation. The germline mutation was inherited from their mother. The hypothesis is that the shared somatic mutation arose in utero in one twin and was then intra-placentally transferred to the other twin [8]. We assume that the somatic mutation in twin A developed after birth, and for this reason, it was not found in both twins.

**Fig. 1:**
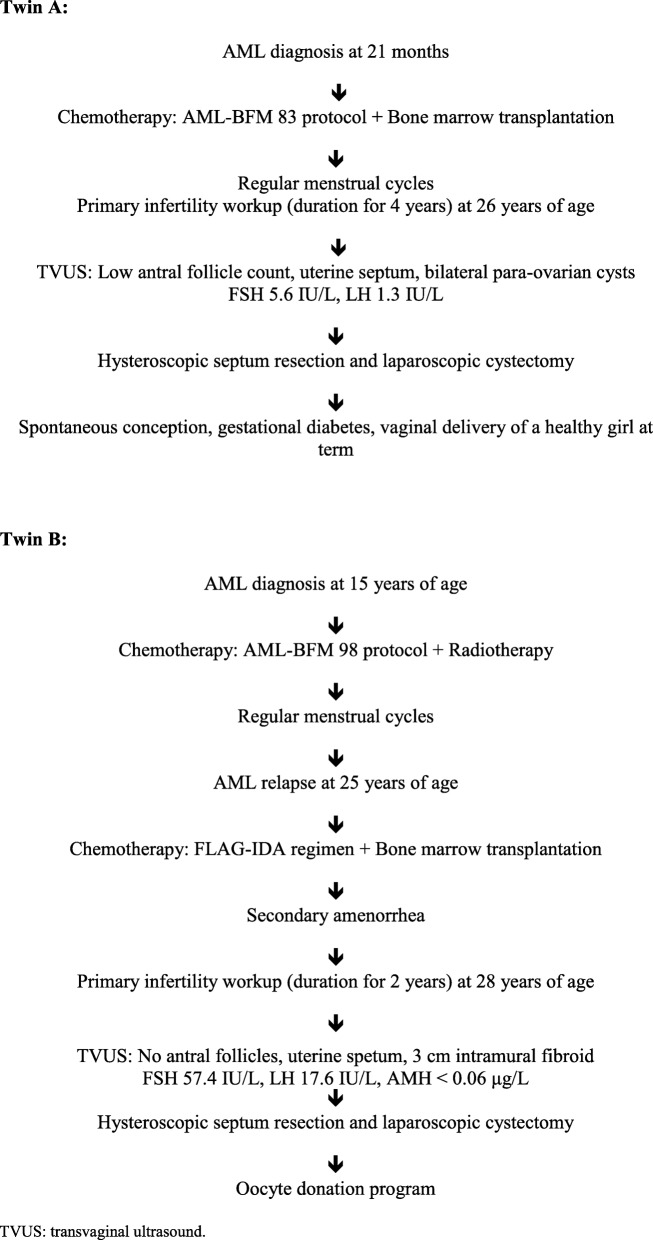
Schematic presentation of the AML and primary infertility diagnostic and treatment process in twin sisters

The menstrual cycle of twin B was regular, every 28–30 days, since she was 12 years-old. Her periods remained regular during and after the AML treatment. However, they became longer in duration and heavier, for which she was prescribed combined hormonal contraceptives. AML relapsed after 10 years, and she received the FLAG-IDA chemotherapy regimen [9] and then underwent BMT from an unrelated donor. She became amenorrhoic after the BMT. The patient was seen at our department because of primary infertility at the age of 28. Her gynecological examination was normal. However, a transvaginal ultrasound revealed an intramural uterine fibroid (Type 4) of approximately 3 cm in diameter located at the uterine fundus and uterine septum. No antral follicles were seen on the ultrasound. Her hormone results were as follows: FSH 57.4 IU/L, LH 17.6 IU/L, AMH < 0.06 μg/L. We performed hysteroscopic septum resection and laparoscopic myomectomy. During laparoscopy, atrophic ovaries were noted. After the surgery, the patient was referred to an oocyte donation program. Figure 1 represents the chronological order of disease treatment and infertility workup in both twins.

## Discussion and conclusions

Twin females in the present case share a common germline N-terminal *C/EBPA* mutation [8] found in 5–14% of AML patients [10]. They also shared a somatic C-terminal *C/EBPA* mutation, which was discovered at the time of the diagnosis and treatment of AML in twin B but was not found later, during her first remission [8]. Furthermore, twin A had an additional mutation in the C-terminal part of the *C/EBPA* gene.

The concordance rate for acute childhood leukemia in MZ twins is between 5 and 25% [2]. The hypothesis of concordance is that leukemia arises in one twin and then spreads to the other twin through the placental vascular anastomoses [11]. In our twin sisters, however, a more plausible explanation for the concordance of AML would be that they share a germline as well as somatic *C/EBPA* gene mutation. The germline mutation was inherited from their mother, whereas the shared somatic *C/EBPA* gene mutation was most probably intra-placentally spread from one twin to the other. Germline mutations of the *C/EBPA* gene increase the risk of the development of AML, whereas somatic *C/EBPA* mutations represent the second event for the development of AML [4]. Lack of the additional somatic mutation in *C/EBPA* gene in twin B, who got sick 13 years after twin A, might explain a longer than usual interval of disease onset.

It is also remarkable that despite the fact that they both had chemotherapy and BMT, only one of them is infertile due to premature ovarian failure.

Cancer treatments constantly improve and many survivors wish to have their own biological children after the treatment conclusion. However, cancer treatments frequently cause infertility [12]. Hence, fertility preservation is crucial for cancer survivors. Fertility preservation options in post-pubertal women include oocyte/embryo cryopreservation, ovarian tissue cryopreservation and GnRH agonist application during the treatment [13], whereas in pre-pubertal girls ovarian tissue cryopreservation is the primary fertility preservation option [14]. Data on the protective effect of the pre-menarchal state on the ovarian reserve are inconclusive [15, 16]. Siris et al. [15] suggested that premature ovarian failure is uncommon in women who were treated with a combination chemotherapy for acute lymphoblastic leukemia (ALL) as children. On the other hand, Quigley et al. [16] observed high FSH levels in girls who survived ALL despite normal pubertal development, suggesting a decreased ovarian reserve. Ovarian function is affected by the age at which a patient undergoes BMT, and undergoing BMT prior to the age of 10 appears to be protective [17]. This seems to be the case for our twin sister A who received chemotherapy and a BMT when she was 2 years-old and is now, 27 years later, fertile. Conversely, BMT after menarche causes infertility in 65–84% of patients [18], and our twin sister B became amenorrhoic and infertile only after the treatment of relapse of AML when she received both chemotherapy and a BMT. Current methods for fertility preservation were unfortunately not available at the time our sisters got sick and were treated for AML.

This is a rare case report of concordant AML in twin sisters. It shows that pre-pubertal chemotherapy obviously does not affect later pubertal development. To the best of our knowledge this is also the only case report describing two different reproductive outcomes after the treatment of AML in monozygotic twin sisters. Despite the fact that spontaneous pregnancies after a BMT are rare and there are data in literature suggesting that only 0.6 to 11% of patients conceive after a BMT [19], our twin sister A, who received chemotherapy and BMT prior to puberty, spontaneously conceived. Twin B, on the other hand, who was treated after menarche, is now infertile. We conclude that although pre-pubertal oncological treatment does not seem to cause immediate infertility, it is important to inform these girls and their parents about future fertility and to offer fertility preservation prior to the start of oncological treatment.

## Data Availability

Not applicable.

## Abbreviations

AML: acute myeloid leukemia
BMT: bone marrow transplantation
